# Implementation Framework to Increase Patient Adherence in Clinical Trials Utilizing Wearable Devices

**DOI:** 10.21203/rs.3.rs-7660915/v1

**Published:** 2025-11-18

**Authors:** Meghan McCarthy, Aubrey Pope, Gabriel Smock, Joshua Kim, Bryan Rettner, Alexander Zhu, Danny Wang, Anna Hamer, Quiana Guo, Allan-Jacob Castillo, Alexandra Potter, Yuhang Zhang, Lizi Zhang, Zhengyu Fang, Xiao Li, Chi-Fu Jeffrey Yang

**Affiliations:** 1 Department of Surgery, Massachusetts General Hospital, Boston MA, USA; 2 Department of Biochemistry, Case Western Reserve University, Cleveland, OH; 3 Center for RNA Science and Therapeutics, Case Western Reserve University, Cleveland, OH; 4 Department of Computer and Data Sciences, Case Western Reserve University, Cleveland, OH; 5 Department of Biomedical Engineering, Case Western Reserve University, Cleveland, OH; 6 Case Comprehensive Cancer Center, Case Western Reserve University, Cleveland, OH

**Keywords:** wearable devices, adherence, clinical trials, digital literacy, implementation strategies, remote monitoring, digital health

## Abstract

**Background:**

Wearable devices are increasingly integrated into clinical trials to collect continuous physiologic data in inpatient, outpatient, and home settings. Consistent patient adherence is essential to ensure high-quality, analyzable data, yet challenges such as digital literacy, device syncing, and socioeconomic disparities persist. Lessons learned from an R01-funded trial at Massachusetts General Hospital, which incorporated wearable devices to extend a machine learning algorithm for monitoring postoperative events after cardiothoracic surgery, offer insight into strategies to improve adherence.

**Methods:**

Insights from the design and implementation of the NIH–NHLBI–funded Wearables Clinical Trial at Massachusetts General Hospital and Case Western Reserve University were synthesized with findings from a focused literature review. This process was used to develop a practical framework of strategies to optimize adherence in clinical trials using wearable devices.

**Results:**

Key strategies include defining clear inclusion and exclusion criteria, offering both virtual and in-person recruitment, reinforcing digital literacy early and repeatedly, establishing regular data monitoring checkpoints, and maintaining targeted participant contact to support accountability. These approaches may enhance participant engagement, reduce data missingness, and promote equitable participation.

**Conclusions:**

Strategies that address technological barriers, participant engagement, and data monitoring can enhance adherence in clinical trials using wearable devices. The proposed framework provides guidance for optimizing study design, improving data quality, and expanding trial accessibility across diverse patient populations. Lessons learned from wearable device integration can inform future studies and facilitate broader adoption of digital health technologies in clinical research.

**Trial registration:**

Not applicable: this article does not report trial results.

## Background:

### Introduction

Wearable devices are technologies worn on the body and often paired with software applications to continuously measure health-related data. According to the U.S. Food and Drug Administration (FDA), they are classified as digital health technologies (DHTs), and certain features within specific devices have been approved as medical devices (Table 1)(1,2).

An estimated 33% of Americans use a wearable device, most commonly smartwatches or health trackers that monitor metrics such as heart rate (HR), saturation of peripheral oxygen (SpO2), and sleep quality(3). More than 80% of users report a willingness to share these biometric data with clinicians, and provider interest in wearable integration has risen significantly(4,5). However, further clinical trials are needed to establish their efficacy and inform best practices. The growing popularity of wearables offers a unique opportunity to expand their role in clinical research.

Wearable devices enable continuous, noninvasive monitoring in naturalistic settings, supporting data collection throughout patients’ activities of daily living (ADLs). Remote monitoring provides several advantages, including objective, real-time measurement and reduced reliance on self-reported information from participants(6). However, trials employing wearable devices depend heavily on participant adherence, particularly consistent device use(7). Adherence may be influenced by factors related to trial design, digital accessibility, and socioeconomic context. Thus, implementation strategies for clinical trials utilizing wearable devices are needed to optimize patient adherence.

While previous studies have examined wearable devices for clinical monitoring, few have systematically addressed the implementation challenges that impact patient adherence in real-world trials. This manuscript uniquely integrates lessons learned from a multicenter, National Institutes of Health (NIH)-National Heart, Lung, and Blood Institute (NHLBI)-funded trial with a structured literature review, providing a practical framework for designing and conducting wearable device-enabled studies. By focusing on actionable strategies, ranging from digital literacy support and virtual recruitment to data monitoring and participant engagement, our work offers generalizable guidance for researchers across disciplines. These insights aim to inform trial designers, clinical researchers, and study coordinators seeking to integrate wearable technologies while maintaining high standards of adherence, data quality, and participant engagement **(Supplemental Table 1).**

### Progress in utilization of wearable devices in clinical trials

Since their introduction to the consumer market within the past decade, wearable devices have been increasingly incorporated into clinical research. These technologies now provide highly sensitive and specific health measures, including real-time single-lead electrocardiogram (ECG) data from devices such as the Apple Watch(22). Concurrently, the growth of the opensource movement has given researchers access to robust datasets and adaptable algorithms to support analysis of these novel data streams(23,24).

Wearables have demonstrated potential across multiple fields of medicine. A landmark trial assessing the Apple Watch’s ability to detect atrial fibrillation enrolled over 400,000 participants, who were monitored for approximately four months(12). Participants with minor arrhythmias received mailed ECG patches for one week of monitoring, while those with major arrhythmias were referred for urgent evaluation. Although not designed to validate the atrial fibrillation detection algorithm, this study established a foundation for large-scale trials to follow.

In endocrinology, wearable automated insulin delivery (AID) systems combine continuous blood glucose sensors with insulin pumps to deliver variable doses in real time (25). Embedded algorithms self-adjust insulin delivery, with data stored in the cloud to allow patients and providers to visualize trends and interact with their health patterns and medication requirements. In psychiatry, wearable devices have been studied for depressive disorders, where objective physiologic measures may complement patient-reported data and reduce the burden of repeated questionnaires(26).

Policy changes have also accelerated wearable adoption in clinical trials. The introduction of new Current Procedural Terminology (CPT) codes in 2019 enabled physicians to bill Medicare for remote physiological monitoring (RPM) (i.e., 99453, 99454, 99457, 99458)(27,28). During the Coronavirus Disease 2019 (COVID-19) pandemic, RPM coverage expanded to include non-chronic conditions and allowed billing for up to 40 minutes per patient per month(29). Over the first year and a half of the pandemic, RPM claims increased by 555%, buoyed by the expanded coverage and benefits associated with remote monitoring(27).

Expansions in RPM reimbursement have facilitated broader adoption of wearable and remote monitoring technologies. Emerging evidence suggests that RPM can improve short-term outcomes, including reduced hospital readmissions and enhanced chronic disease management, particularly when monitoring is paired with timely provider review and intervention(30–32). However, results are heterogeneous, and increased billing alone does not guarantee improved outcomes. Patient adherence, engagement with the monitoring devices, and actionable follow-up are critical determinants of effectiveness(30–32). Long-term data evaluating impacts on morbidity, mortality, and patient-reported outcomes remain limited.

### Challenges to implementing wearable devices in clinical trials

Despite their broad applications, several challenges hinder the integration of wearable devices into clinical trials. While wearables can provide an inexpensive health monitoring tool, participation often requires smartphone ownership and reliable internet access. These prerequisites may disproportionately exclude patients from lower socioeconomic backgrounds. For example, although 97% of adults aged 18–49 own a smartphone, ownership declines to 76% among adults aged 65 years and older(33). Similarly, 98% of adults in households earning $100,000 or more annually own a smartphone, compared to 79% in households earning less than $30,000 per year(34). These disparities in access can create significant barriers to enrolling participants from diverse socioeconomic groups(35).

Digital literacy further impacts data quality once participants are enrolled. Digital literacy refers to skills that allow individuals to understand, integrate, and even analyze digital tools and can depend on my demographical factors as outlined in the challenges section. Most wearable devices rely on Bluetooth syncing with smartphones to enable remote, continuous monitoring. Confidence and comfort with technology, often referred to as the “digital divide,” varies substantially across age groups(36). Older adults may face challenges due to limited prior experience or lower technology skills(37). These barriers are amplified among some racial and ethnic minority groups(36). For example, previous research has demonstrated that Black and Hispanic patients are less likely to use wearable devices compared to White counterparts due to factors such as limited technology access, lower digital literacy, and mistrust in healthcare systems(38).

Adherence to device use also presents a significant challenge. Remote monitoring shifts responsibility to participants, who may struggle to maintain consistent wear during travel, illness, or stressful life events(39). Breaks in adherence can result in substantial data loss, undermining study validity(40). Although scheduled monitoring checkpoints and outreach protocols may help, participant engagement ultimately determines adherence to protocol.

Finally, the use of wearable devices and companion applications raises concerns about privacy and data security(7). These devices collect vast amounts of personal health data, including heart rate, activity levels, and sleep patterns, often stored in the cloud and accessible to third parties. Such data handling practices raise concerns about unauthorized access and potential breaches. For instance, a study evaluating the privacy policies of seventeen leading wearable technology manufacturers found that many lacked transparency, data minimization, and user control over their information(41). Moreover, the regulatory landscape for wearable devices is still evolving, with concerns about compliance with data protection laws like the Health Insurance Portability and Accountability Act (HIPAA). Unclear data sharing practices may deter enrollment, and safeguarding participant information requires explicit consent processes, robust patient education, and input from privacy specialists during trial design.

## Methods

### Study Context and Design

The Wearables Clinical Trial at Massachusetts General Hospital (WCT-MGH) is a multicenter study funded by the NIH–NHLBI and approved by the Massachusetts General Brigham Institutional Review Board (Protocol #: 2020P002984; Clinical Trial #: NCT04824066). The study was designed to extend a previously validated machine learning algorithm for the early detection of abnormal physiological events, including postoperative complications following cardiothoracic surgery. While this manuscript does not report trial outcomes, the WCT-MGH protocol provides a model for implementing wearable devices in clinical trials and contextualizes lessons learned to improve participant adherence.

Eligible participants included adults undergoing cardiac or thoracic surgery at participating centers. Patients were recruited either in person during preoperative visits or virtually via telephone. Verbal informed consent was obtained from all participants prior to enrollment. Following verbal consent, participants are asked to download the Fitbit (Fitbit, Inc.) mobile application and are provided with a Fitbit Charge 4, 5, or 6. Study staff assist with device setup and verify successful syncing of biometric data. Patients are instructed to wear the device for at least one week prior to surgery and for at least 90 days postoperatively. Before hospital discharge, study staff revisit participants to answer questions, troubleshoot device use, and reinforce digital literacy. Biometric data are reviewed weekly through investigator-led audits to ensure transmission and integrity. Patients also receive daily surveys delivered through the Case Western Reserve University (CWRU Heartbit app and follow-up questionnaires via the Research Electronic Data Capture (REDCap) email(43,44). Direct outreach occurs only if a participant fails to sync their device or complete surveys for more than one week.

Since its pilot phase in 2021, the clinical and data analytics teams have iteratively refined the study protocol, resulting in multiple Institutional Review Board (IRB) amendments aimed at optimizing trial design and participant adherence. These refinements, informed by practical challenges and ongoing evaluation, laid the foundation for the framework detailed in the Results section.

### Literature Review

To complement trial experience, a targeted literature review was conducted using PubMed for peer-reviewed publications through 2025. Search terms examples included *wearable devices, clinical trials, adherence*, *compliance*, *digital health*, and *clinical trials*. Articles were considered eligible if they discussed clinical trials utilizing wearable devices, patient adherence, study implementation, or barriers to wearable device use in clinical or research settings. Additional relevant studies were identified through citation chaining.

### Development of Implementation Strategies

Findings from the trial and literature review were synthesized through iterative discussions among study investigators. Challenges and corresponding solutions were grouped into thematic domains relevant to patient adherence in wearable device trials. These include recruitment and eligibility criteria, digital literacy and participant training, data monitoring and quality control, participant engagement and retention, and equity and accessibility. These domains form the foundation of a structured implementation framework for wearable device-enabled trials. Within each, specific strategies were developed to optimize adherence, promote high-quality data collection, and ensure inclusive participation. This structured framework reflects both practical lessons learned from trial operations and broader evidence from published studies, providing a generalizable guide for researchers implementing wearable device-based clinical trials.

## Results: Implementation Framework for Adherence

Insights from the Wearables Clinical Trial and literature review were synthesized into a practical implementation framework to address adherence in wearable device–enabled studies. Five key domains were identified: (1) determining sample size, (2) defining inclusion and exclusion criteria, (3) offering virtual flexibility in recruitment and participation, (4) establishing researcher–participant rapport, (5) monitoring data quality, and (6) promoting digital literacy and accessibility. Within each domain, targeted strategies were developed to mitigate common adherence challenges and enhance participant engagement, data quality, and workflow efficiency. A summary of these domains, their associated challenges, strategies, and benefits is presented **(Supplemental table 2)**.

### Determining sample size

Optimal sample size in clinical trials utilizing wearable devices depends heavily on study goals and directly influences the feasibility and success of implementation. Several easy-to-use software tools are available to calculate sample size for randomized controlled trials (RCTs) *a priori*, minimizing both type I and type II errors(45). However, practical considerations, such as available resources, recruitment capacity, and study aims, often shape the final decision. In the WCT-MGH, for example, the enrollment target is 1,200 participants over five years, translating to a weekly recruitment goal of approximately 4.8 patients.

While larger sample sizes increase statistical power, they also present tradeoffs. Expanding enrollment can raise the risk of loss to follow-up, which may reduce generalizability. Screening and verifying eligibility become more labor-intensive, and participants may misclassify themselves as eligible despite not meeting criteria(12). Larger cohorts also increase financial burden due to the cost of supplying wearable devices, and generate greater demands for data wrangling, storage, and interpretation(46). For research teams already overburdened, pursuing a more modest sample size may be more realistic and sustainable.

Conversely, smaller sample sizes are more feasible but inherently limit statistical power. Single-center trials with smaller cohorts are easier to coordinate, though their findings may not be conclusive in isolation(47). However, a series of smaller trials can be combined through metaanalysis to produce more robust conclusions. Importantly, smaller cohorts allow research staff to devote more time to providing technical support, such as tedious device setup and troubleshooting, which can be critical for maximizing adherence.

Ultimately, determining sample size in wearable device–enabled clinical trials requires balancing statistical power, feasibility, and the resources available to sustain participant engagement and data quality.

### Defining inclusion and exclusion criteria

Clear inclusion and exclusion criteria are essential when designing clinical trials that utilize wearable devices. These decisions must account for the many factors that influence biometric data quality and interpretation. For example, in the WCT-MGH, biometric data from cardiac surgery patients is frequently confounded by the postoperative use of beta-blockers. AV nodal blocking agents such as metoprolol can alter heart rate variability (HRV) in ways that complicate interpretation of wearable derived HRV, a key endpoint for the study(48,49). To address this, WCT-MGH investigators amended the study protocol to include surveys tracking both pre- and postoperative medication use. In surgical populations more broadly, criteria should also reflect the expected prevalence and type of postoperative complications, as these can affect both outcomes and adherence.

Beyond surgical settings, eligibility criteria must consider how pre-existing health conditions, age, race and ethnicity, and sex may shape biometric data. A 2016 meta-analysis demonstrated that women have a higher mean heart rate than men but are not at higher risk for cardiovascular disease(50). A secondary analysis in 2015 further suggested that African Americans have greater HRV than European Americans, even after adjusting for covariates(51). For trials such as the WCT-MGH, where HRV is central to algorithm development, these population-level differences are critical to consider.

Eligibility criteria also directly intersect with sample size. Stricter criteria narrow the recruitment pool but simplify data interpretation by reducing confounding variables such as postoperative beta-blocker use. Broader criteria enhance generalizability but introduce greater variability in biometric data. Clinical researchers must weigh the advantages and disadvantages of including criteria to ensure desired statistical power and generalizability while the biometric data remains coherent.

### Offering virtual flexibility

Designing clinical trials utilizing wearable devices with virtual participation offerings may benefit patient recruitment, adherence, and retention. In the WCT-MGH, cardiac patients are typically recruited and set up after their preoperative in-person clinic visit. Many thoracic patients, however, have their preoperative appointments conducted via telehealth. To accommodate this workflow difference, WCT-MGH staff members call eligible thoracic surgery patients for recruitment, ship wearable devices directly to participants, and then assist with device setup virtually.

Broader literature suggests that virtual recruitment and decentralized study designs can expand reach and reduce barriers to participation. After telemedicine appointments and policies expanded in response to the COVID-19 pandemic, this essential offering continues to remain in many clinical settings(52). Remote recruitment and retention strategies have been shown to improve accessibility for patients, minimize disruption to clinical workflows, and reduce loss due to missed in-person visits ((53,54) Providers value its enhancement of health literacy and workforce expansion through virtual education and training (55).

Virtual flexibility also mitigates long-standing challenges such as patient no-shows, which can range from 2–30% in outpatient settings(56). Patient no-shows are a long-standing issue which affect resource utilization, workflow, and quality of healthcare services(57). In the WCT-MGH, calling patients shortly after a missed preoperative visit enabled successful enrollment that might otherwise have been lost. Furthermore, studies suggest that even when survey completion declines, participants often continue to share passive wearable data, indicating that virtual participation can sustain data collection over longer periods(58). Lastly, clinical trials designed with an option of virtual flexibility offer a backup infrastructure in the event of understaffing or unpredictable conflicts.

### Establishing researcher-patient rapport

Whether establishing rapport between researchers and participants is appropriate in a clinical trial requires careful consideration(59). While rapport may enhance patient responsiveness and adherence to study protocols, it can also introduce social desirability bias, potentially affecting data quality(60). In trials centered on quantitative outcomes such as biometric data, however, building rapport can be advantageous. Wearable-based trials often involve frequent participant–researcher interactions due to device setup, troubleshooting, and syncing requirements.

Developing consistent relationships with study staff can minimize confusion and maximize adherence to protocol. Patients may feel uncomfortable or disengaged when interacting with multiple coordinators, whereas rapport can increase motivation, engagement, and willingness to resolve technical issues(61). Large-scale analyses of digital health studies reinforce that convenience, clinician involvement, and early support are key to sustaining participant engagement(62,63).

In the WCT-MGH, participant adherence to wearing and syncing devices depends not only on protocol compliance but also on timely collaboration with study staff when technical difficulties arise. Participants minimally interact with the team at four major points: recruitment, setup, postoperative inpatient check, and study completion. Additional outreach occurs if weekly audits reveal missing data. The study team has observed that patients are more responsive and consistent in syncing their devices when they maintain a single, consistent point of contact throughout participation. Conversely, patients often report confusion when the staff member who recruited them is different from the one who follows them postoperatively. To mitigate this, WCT-MGH aims to pair participants with a designated staff member for the duration of the trial. This approach has fostered stronger engagement, with participants proactively reaching out to their coordinator for assistance about even optional device features such as the ECG monitor.

### Monitoring data quality

In clinical trials utilizing wearable devices, data quality can be improved through the development of quality checks (QCs) and monitoring infrastructure. Complete datasets rely on participants both wearing their devices and meeting syncing requirements. Because these requirements vary across devices, trials often face issues of “data missingness,” a well-recognized challenge arising from device non-wear, failure to sync, battery drains, or data transmission issues (64). Data completeness is typically measured by the availability of daily heart rate and aggregate metrics(65). Participants frequently encounter syncing challenges due to forgetfulness, technological obstacles, competing demands of daily life, or lapses in communication with the research team. Clear and consistent communication about proper device-phone syncing is therefore critical.

When designing wearable trials, teams should establish a priori thresholds for data completeness to determine whether a participant’s data is usable. In the WCT-MGH, this threshold is defined as 0.33, or at least 8 hours of heart rate data per day. To detect and remediate lapses in syncing or data collection, the WCT-MGH employs a weekly QC process. After enrollment and successful device-phone pairing, participants are instructed to sync their Fitbit once per day. Because the Fitbit Charge 4/5 (5/6) stores data for up to 5 days, weekly surveillance is sufficient. Each week, the MGH team provides a de-identified list of all active study participants to CWRU, which verifies syncing at least once within the past 7 days. Syncing status is confirmed using the Fitbit Dashboard, and each participant is labeled as either “active” or “inactive.” Individuals who have not synced their devices within a seven-day timeframe are designated as “inactive” participants.

Participants labeled as inactive receive follow-up communication via text or telephone early in the week to allow ample time for re-syncing. Successful syncing is then confirmed through CWRU. If a participant remains unresponsive and does not sync their device for two consecutive weeks, their QC monitoring is discontinued.

While QC protocols may vary by study in terms of frequency, thresholds, and specific workflows, establishing clear monitoring policies is essential. Implementing structured QCs can minimize data missingness, thereby improving both adherence and overall data quality within the cohort.

### Promoting digital literacy and accessibility

Because participation in wearable device studies often requires smartphone ownership, internet access, and comfort with technology, clinical trials must proactively support participants with varying levels of digital literacy. Rather than serving as an additional barrier, study design should incorporate infrastructure that enables participants across age, racial, and socioeconomic backgrounds to engage equitably. There are many implications from this “digital divide” in clinical trials utilizing wearable devices. Studies working with older adults and minorities must be mindful that device setup, syncing, and other functionalities may not be intuitive or easy for study participants.

To address this, the WCT-MGH has incorporated multiple supports into its protocol. All study staff are trained on Fitbit functionality, including setup, troubleshooting, and problem-solving common malfunctions. During in-person recruitment, staff work directly with participants to set up the device, download the Fitbit app, and complete Bluetooth pairing. For patients recruited virtually, staff schedule a follow-up call once the device arrives to guide them step-by-step through the process. At the postoperative visit, staff again review device use and address any questions. This real-time technology support is essential to the success of virtual recruitment for the WCT-MGH.

To further promote independence and engagement, WCT-MGH developed patientfriendly resources, including short instructional videos demonstrating how to set up, sync, and use features of the Fitbit. These can be shared directly or accessed via the WCT-MGH website, ensuring participants have reference materials outside of scheduled touchpoints. Throughout participation, patients also have access to staff for ongoing technical support.

Evidence supports the effectiveness of these strategies. Tailored digital literacy interventions improve older adults’ confidence and independence when using telehealth and mobile health technologies(66). Technical assistance is frequently necessary, as older adults often face difficulties maintaining device use over prolonged monitoring periods(67). Community-based training programs for underserved older adults have demonstrated that structured teaching and digital mentorship can enhance adoption and self-efficacy with digital health tools(68). Furthermore, peer and professional support models increase adherence to wearable monitoring by providing ongoing encouragement and troubleshooting assistance(69).

Ultimately, investing in digital literacy infrastructure not only improves adherence and data quality but also enhances equity in trial participation. Studies that intentionally bridge the digital divide are better positioned to include older adults and underserved groups, increasing the generalizability and impact of their findings.

## Discussion

The implementation framework developed in this study highlights that successful clinical trials using wearable devices depend on participants’ consistent engagement to ensure high-quality data collection. While challenges such as digital literacy, device syncing, and data completeness persist, careful study design can mitigate these barriers. Trials that employ well-defined inclusion criteria, offer virtual flexibility, establish data monitoring protocols, foster researcher-participant rapport, and assess digital accessibility tend to achieve higher adherence.

This study has several limitations. First, as an implementation-focused manuscript, it does not report empirical trial outcomes. The framework presented is primarily derived from experiences within a single multicenter trial and a targeted literature review, which may limit generalizability. Second, qualitative insights from study staff and participant interactions could be influenced by observer bias or selective reporting. Finally, rapid evolution of wearable technology may outpace specific recommendations, requiring ongoing adaptation of protocols. Despite these, the framework does provide a practice and evidence informed foundation for increasing adherence in future wearable device-enabled trials.

Beyond biometric data collection, wearable devices show promise in promoting positive health behaviors. Studies demonstrate that real-time feedback on vitals, sleep, and activity can encourage both prompted and self-directed improvements in lifestyle and functional recovery(70). For example, wearable-assisted rehabilitation programs in stroke patients have been associated with enhanced functional outcomes(71). Wearable devices may help manage both acute and chronic disease. While the WCT-MGH focuses on detection of postoperative complications prior to symptom onset, wearables are frequently used in cardiovascular medicine for remote management of chronic conditions such as heart failure and peripheral artery disease(72).

Wearables also have implications for prevention, detection, and management in clinical care. Continuous and remote monitoring enables timely identification of abnormal physiological events and patterns, while features such as irregular rhythm notifications and on-demand ECGs can facilitate provider engagement (46). Both Apple Watch and Fitbit devices offer features to share health data and report results with providers. Surveys indicate that a majority of adult users are willing to share wearable data with clinicians, highlighting their potential to integrate seamlessly into patient care models(4).

The experience from WCT-MGH provides a practical framework for implementing wearable devices in clinical trials. By incorporating weekly data monitoring, virtual setup support, structured technical resources, and consistent staff-participant contact, the study demonstrates strategies to optimize adherence and data quality in a surgical population. Lessons learned from WCT-MGH illustrate that successful integration of wearable devices requires not only technological innovation but also deliberate attention to participant support, accessibility, and engagement.

## Conclusion

As wearable technology evolves, integrating these tools into clinical trials and healthcare delivery presents opportunities to improve both research quality and patient outcomes. Future efforts should focus on refining implementation frameworks that are inclusive, adaptive, and centered on the participant experience. Ultimately, the framework presented underscores that the impact of wearable devices will depend not only on technological advancement but also on an ability to translate these advances into equitable, effective, and patient-centered health solutions.

## Figures and Tables

**Figure 1:**
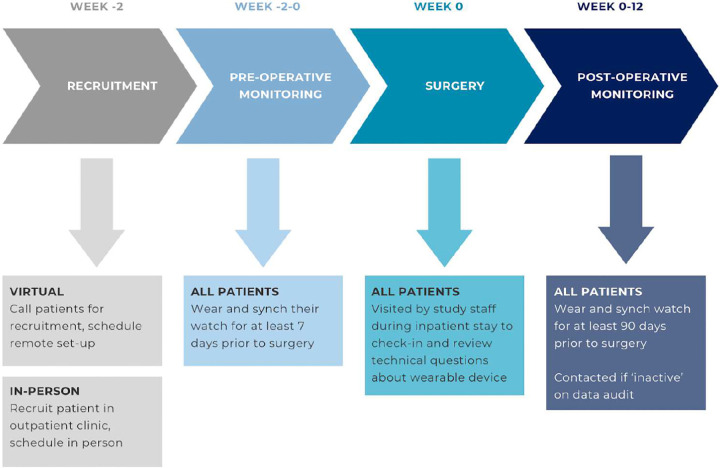
Workflow of WCT-MGH participation Participation checkpoints in the WCT-MGH

## Data Availability

Data supporting the findings of this study are available from the corresponding author upon reasonable request.

## List of abbreviations

ADLs: Activities of Daily Living
AID: Automated Insulin Delivery
CPT: Current Procedural Terminology
COVID-19: Coronavirus Disease 2019
CWRU: Case Western Reserve University
DHTs: Digital Health Technologies
ECG: Electrocardiogram
FDA: U.S. Food and Drug Administration
Fitbit: Fitbit, Inc.
HIPAA: Health Insurance Portability and Accountability Act
HR: Heart rate
HRV: Heart Rate Variability
IRB: Institutional Review Board
MGH: Massachusetts General Hospital
NIH: National Institutes of Health
NHLBI: National Heart, Lung, and Blood Institute
QC: Quality Check / Quality Control
REDCap: Research Electronic Data Capture
RPM: Remote Physiologic Monitoring
RCT: Randomized Controlled Trial
SpO2: Saturation of Peripheral Oxygen
WCT-MGH: Wearables Clinical Trial at Massachusetts General Hospital
